# Practical Notes and Formulæ

**Published:** 1887-07

**Authors:** 


### PRACTICAL NOTES AND FORMULÆ.

**Bloody Flux.**—If the bloody discharges have not been preceded by diarrhœa, I give:

℞. Magnesiæ Sulph.................................℥ j,

Tinct. Opii.....................................ʒ j,

Aquæ.............................................℥ iv.

M. Sig—One-fourth every three hours until it moves the upper bowels.

Then stop it and give:

℞. Bismuth subnit.................................ʒ j,

Dover's powd......................................ʒ j.

M. and make twelve powders. Sig.—Give one every four hours until better, then as necessary.

In connection with the second prescription, I give:

℞. Alum...........................................ʒ ij,

Aquæ.............................................pints ij.

M. Sig.—Inject into the bowels every six hours.

When the above injection is returned by the bowels, I give injection of starch and laudanum, and tell the patient to retain it as long as he can.

If diarrhœa precedes the bloody discharges, I leave off the first prescription, and begin at once with the second one. Of course, poultices will come in all along the line of treatment.—*Dr. Deason in Medical Brief.*

**Diabetis Mellitus.**—But perhaps the most successful treatment is diet. Bran bread, milk, eggs, whey, coffee and tea; beef, mutton, fish, birds, soups without flour, cabbage, spinach, celery, lettuce, onion—crabs, lobsters and oysters. Attention should be paid to the skin, keeping up the action and giving physiological rest to the kidneys; hence, the vapor bath is very useful.

By dieting the patient and using the following formulas, we have cured several cases:

℞. Ext. pilocarpi.................................fl℥ iv,
Rhus aromaticæ...................................℥ iv,
Ext. ptelea trifol...............................fl℥ iv.

M. Sig.—Teaspoonful every three hours.

℞. Acidi sulph. arom..............................ʒ j,

Tinct. ferri perchlor............................ʒ j,

Inf. gentian comp................................℥ iv.

M. Sig.—Teaspoonful every three hours, or alternate with the two.

To relieve thirst, milk and lime-water, or chloride potash in milk diluted with water; take a sip when needed, but liquids had better be avoided as much as possible.—*Medical Brief.*

**For Dysentery.**—

℞. Leptandrin (trit.)................................gr. x,
Carbo vegetablis.........................................gr. v.

M. Sig.—At a dose every two hours.

℞. Quiniæ sulph........................................gr. v,
Bismuth subnit..........................................gr. v.

M. Sig.—At a dose, every two hours. Alternate with the above.—*Brief.*

**Camphor Vapor in Bronchitis.**—A writer in *Eclectic Medical Journal* say: The method of treatment is simple. Saturate a small sponge—or a handkerchief will answer very well—with spirits camphor, and inhale by holding over the mouth when there is any inclination to cough. The patient should inhale cautiously at first, as the inhalation will be followed by what appears to be an increased irritation and desire to cough, but by a little perseverance this will rapidly disappear. Repeat the application as often as the desire to cough returns. A mild tonic may be given if necessary. I believe speedy recovery in nine cases out of ten will result from the above mode of treatment.

**For diarrhœa** of three months' duration, characterized by a desire to evacuate the bowels immediately after eating, Prof. Bartholow advises the following plan of treatment: Put patient on a milk diet as far as possible, also—

℞. Creasoti...........................................*m* ij,
Bismuthi subcarb...................................gr. x–xv,
Glycerin..............................................f ℥ ss.
M. Sig—Ter die, before meals.—*Clinical Record.*

**The Treatment of Colds.**—Dr. J. H. Whelan states in the *Practioner* for March, 1887, that he has found a combination of belladonna, quinine, and arsenic almost specific in aborting common colds if commenced in the early stage of the affection, while it is still confined to the nose and pharynx. The formula which he uses is the following:

℞. Quininæ sulphatis..............................gr. xviii,
Liquoris arsenicalis..............................*m* xii,
Liquoris atropinæ.................................*m* i,
Extracti gentianæ................................gr. xx,
Pulveris gummi acaciæ, q. s. ut fiant pilulæ..xii.

Sig.—One every three, four, or six hours, according to circumstances.

Dr. Whelan states that at starting one pill should be taken every three or four hours, and later on every six hours, and he believes that if a catarrhal subject has a box of these pills always at hand he will almost invariably succeed in aborting a cold.

He does not profess to explain how his remedy acts, unless it be as a powerful nervine and general tonic.—*Therapeutic Gazette.*

**The Treatment of Stye.**—Styes are such troublesome little ailments that the following remedy for their cure, recommended by M. Abadil, may be welcome:

℞. Acidi boracic........................grammes x,

Aquæ dest...........................grammes ccc.

Dissolve.

With a wetted piece of wadding, drop some of this solution on the stye several times a day. It is said not only to effect a cure, but to prevent a return of the annoyance.—*Exchange.*

**Constipation of Children.**—A very successful remedy for this is podophyllin, in small doses; iridin may be combined with it with goood effect. Make a tincture of the following:

R. Podophyllin. resin.........................gr. viij,
Iridin.................................gr. v,
Spirit. ammon. arom.........................f ℥ j.

Digest for several days, and filter.

One or two drops of this may be given at bedtime on a small piece of loaf sugar, or the dose may be combined in mixture along with syrup of orange. This is the dose for a child of one year and under.—*Clinical Record.*

**Ointment for the Relief of Ocular Pains.**—For the relief of severe orbital pain following iritis, hyperæsthesia of the retina and neuralgia of the eyeballs, Dr. L. Webster Fox, of Philadelphia, prescribes the following ointment, which, he informs us, he has found to be of exceptional utility:

℞. Morphiæ sulph..............................gr. iv,
Chloral...................................gr. x,
Cocaine...................................gr. xx,
Menthol...................................gr. xxx,
Lanolin...................................ʒ j.

M. Sig.—Apply a piece the size of a hazel nut to the temple and over the brow every hour.—*Exchange.*

**Glycein in Phthisis**.—Prof Jaccoud, of the Paris Faculty, has strongly recommended (*La Therapeutique Contemporarie*) the use of glycerine as an excitant and agent d'epargne, during the non febrile period of common phthisis, when cod liver oil is no longer tolerated. He recommends the following mixture, which should be taken in the course of the day, either with or between meals:

℞. Rum or cognac..............................grams x,

Ol. menth. pip..............................♏ j,

Glycerin..................................grams xl.

M. This mixture is agreeable to the taste and well tolerated by the stomach; even after several months, it induces neither satiety nor disgust. The quantity is sufficient for one day, but in cases which present no sign of abnormal excitability of the nervous system or heart, the dose of glycerine may be gradually raised to 50 or 60 gram per diem.—*Medical and Surgical Reporter.*

